# Placental pathology and neonatal encephalopathy

**DOI:** 10.1002/ijgo.14301

**Published:** 2022-07-03

**Authors:** Aine Fox, Emma Doyle, Michael Geary, Breda Hayes

**Affiliations:** ^1^ Department of Neonatology The Rotunda Hospital Dublin 1 Ireland; ^2^ Royal College of Surgeons Ireland Dublin 2 Ireland; ^3^ Department of Histopathology The Rotunda Hospital Dublin 1 Ireland; ^4^ Department of Obstetrics and Gynaecology The Rotunda Hospital Dublin 1 Ireland

**Keywords:** cerebral palsy, fetal vascular malperfusion, hypoxic–ischemic encephalopathy, neonatal encephalopathy, placenta

## Abstract

Neonatal encephalopathy (NE) is an important cause of neonatal morbidity and mortality worldwide; however, there remain gaps in our knowledge about its pathogenesis. The placenta has been implicated in the pathogenesis of this disease but conclusive evidence related to the placental factors that influence it is sparse. This review aims to outline the current knowledge on the role of the placenta with particular attention to its role in NE as a consequence of hypoxia‐ischemia. A total of 26 original articles/review papers were used to compile this review. Three themes were identified from these publications: fetal vascular malperfusion including umbilical cord pathology, inflammatory changes in the placenta, and maternal vascular malperfusion including placental weight. These features were identified as being significant in the development of NE. Advancing our understanding of this relationship between placental pathology and NE may facilitate the development of additional antenatal screening to better identify at‐risk fetuses. We highlight areas for further research through antenatal screening and placental histology.

## Synopsis

This article examines the association between neonatal encephalopathy and placental pathology. It highlights the specific pathology implicated in the pathogenesis of neonatal encephalopathy.

## 1 BACKGROUND

Neonatal encephalopathy (NE) in the term or near‐term infant is a condition of disturbed neurological function.^1^ The incidence of NE is estimated at approximately three in 1000 births in high‐income countries^2^, ^3^ and up to 26 in 1000 births in low‐middle–income countries.^4^

Hypoxic–ischemic encephalopathy (HIE) is often used interchangeably with NE; however, this term exclusively refers to NE as a consequence of hypoxia‐ischemia.^5^ In reality it is not always possible to determine the exact cause of NE often because an incomplete clinical picture further compounds the gaps in medical knowledge and research in this area.^3^ The clinical features of NE are categorized into grades of severity using a recognized scoring system. The grade of NE is used to aid prognostication and guide clinical management, including identifying infants who may benefit from currently available neuroprotective treatments. The Sarnat scoring system was first described in 1976 and has been modified for this purpose.^6^ Unfortunately, the parameters used to identify infants with acute hypoxia are nonspecific and can also be present in chronic hypoxia, making the distinction between the two entities difficult.^7^

For most pregnancies, the placenta functions effectively to facilitate appropriate fetal growth and development during pregnancy. The placenta should support the safe delivery of the baby through the normal physiologic hypoxic environment of labor.^5^ It is estimated that 23% of neonatal deaths worldwide occur because of intrapartum complications,^5^ and these outcomes are likely to be influenced by placental factors.^2^, ^8^

A proportion of pregnancies result in an acute catastrophic event, such as placental abruption, uterine rupture, shoulder dystocia, or cord prolapse, leading to hypoxia‐ischemia for the fetus. Many more are subjected to a more gradual and prolonged hypoxia‐ischemia. It is estimated that ≈75% of the cases of babies who experience hypoxia‐ischemia are a result of a gradual prolonged hypoxic environment as a consequence of uterine contractions.^5^ It is not clear why fetuses with the same apparent labor history can have dramatically different outcomes. The placenta has long been considered the “blackbox” of pregnancy. Pathology of the placenta is thought to double the risk of NE,^9^ but how specific placental pathologies contribute to this remains poorly understood. Previously, researchers identified the increased incidence of placental pathology for babies born with NE as compared with well babies.^2^ Placental pathology is a double‐edged sword. It can cause brain injury before delivery, while also making the well fetal brain vulnerable to injury during labor and delivery.^1^, ^10^, ^14^

It is common practice to examine the placenta of babies born with NE or who are stillborn.^9^ This practice has been recommended by many professional bodies including the Royal College of Obstetricians and Gynecologists and the Royal College of Pathologists in the United Kingdom since 2001.^11^ This practice is to investigate the cause of NE for each case. Historically, the approach to placental examination and reporting has varied between pathologists, making it difficult to compare placental findings and neonatal outcome. In 2016, the Amsterdam Placental Workshop Group developed a consensus statement outlining standardized guidelines for examining and reporting placental findings.^12^, ^13^ This is to aid streamline reporting of placental pathology.^16^ It defines umbrella terms for reporting lesions previously reported in isolation.

Better understanding of how placental pathology contributes to the development of NE may aid earlier recognition of the at‐risk fetus. It will promote the development of new treatment strategies to prevent or ameliorate this potentially devastating condition. This review article aims to highlight the pathological features associated with NE.

## 2 MATERIALS AND METHODS

A literature review was performed using the MEDLINE database. Results included articles and review papers published between January 1, 2006, and July 2021. The search was performed on July 15, 2021, and included a search of mesh terms and key words. The keywords search included: “placenta,” “infant,” “encephalopathy,” “cerebral palsy,” “HIE,” and “hypoxic ischemic encephalopathy.” Only English‐language articles were reviewed.

The Amsterdam International Consensus group of placental pathologists published guidelines for formal reporting. This review followed these guidelines for describing findings.

Fetal vascular malperfusion (FVM) describes fetal vascular obstructive lesions, fetal thrombotic vasculopathy (FTV), fetal vascular thrombi, and extensive avascular villi.^16^

Maternal vascular malperfusion (MVM) describes lesions that impair maternal placental circulation, previously referred to as maternal vascular underperfusion^16^ or chronic uteroplacental insufficiency. MVM includes gross (eg, placental hypoplasia, infarction, and retroplacental hemorrhage) and microscopic (eg, distal villous hypoplasia and accelerated villous maturation) features.

## 3 RESULTS

**FIGURE 1:**
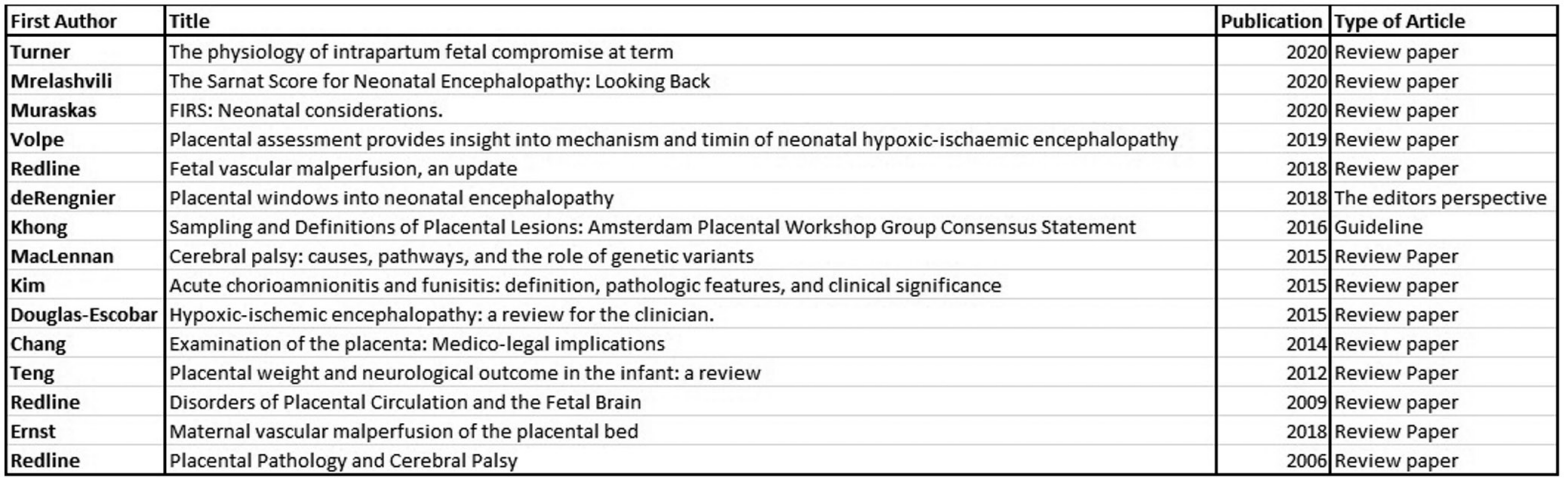
Review articles reviewed.

**FIGURE 2:**
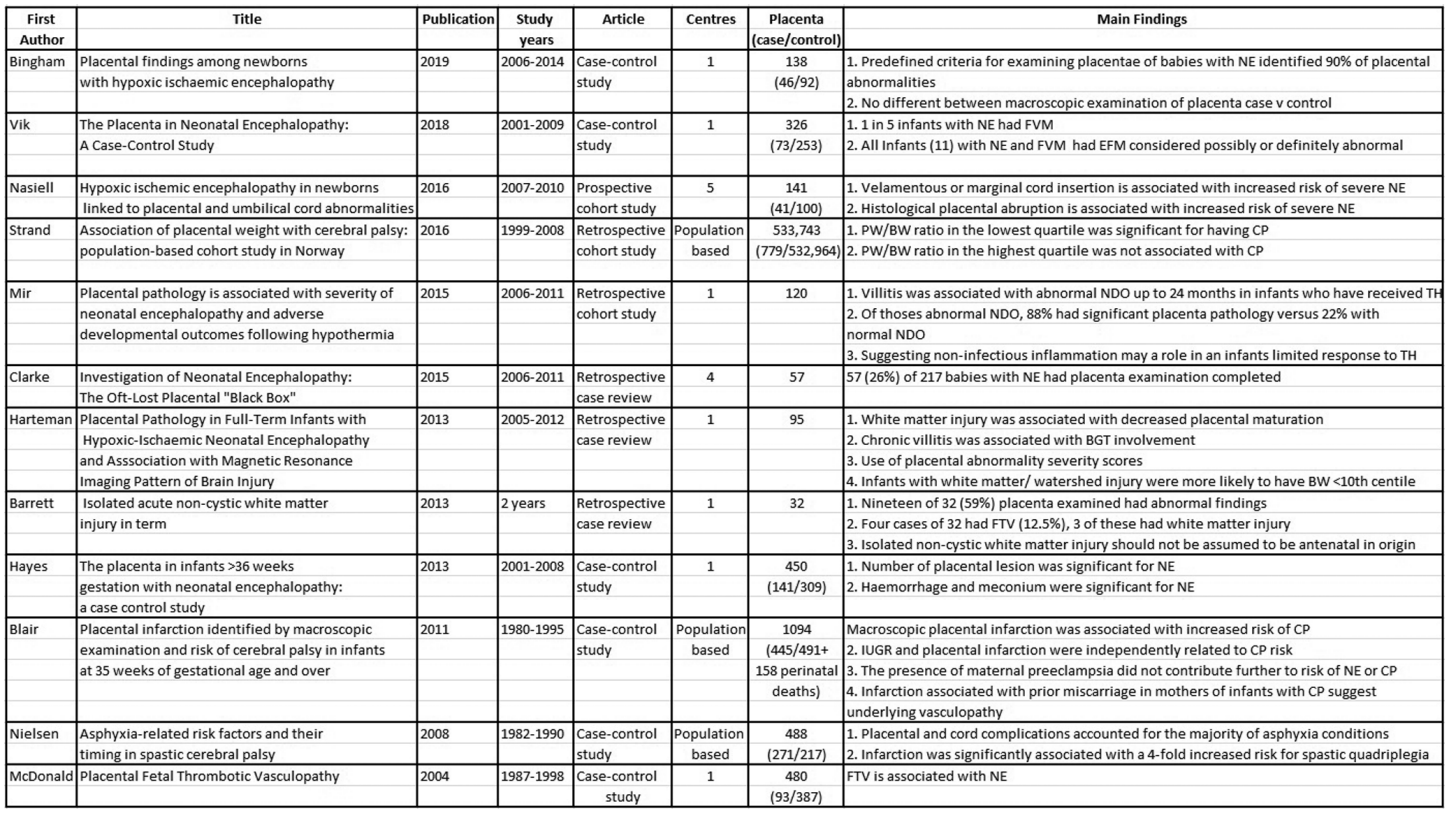
Original research articles reviewed.

**FIGURE 3:**
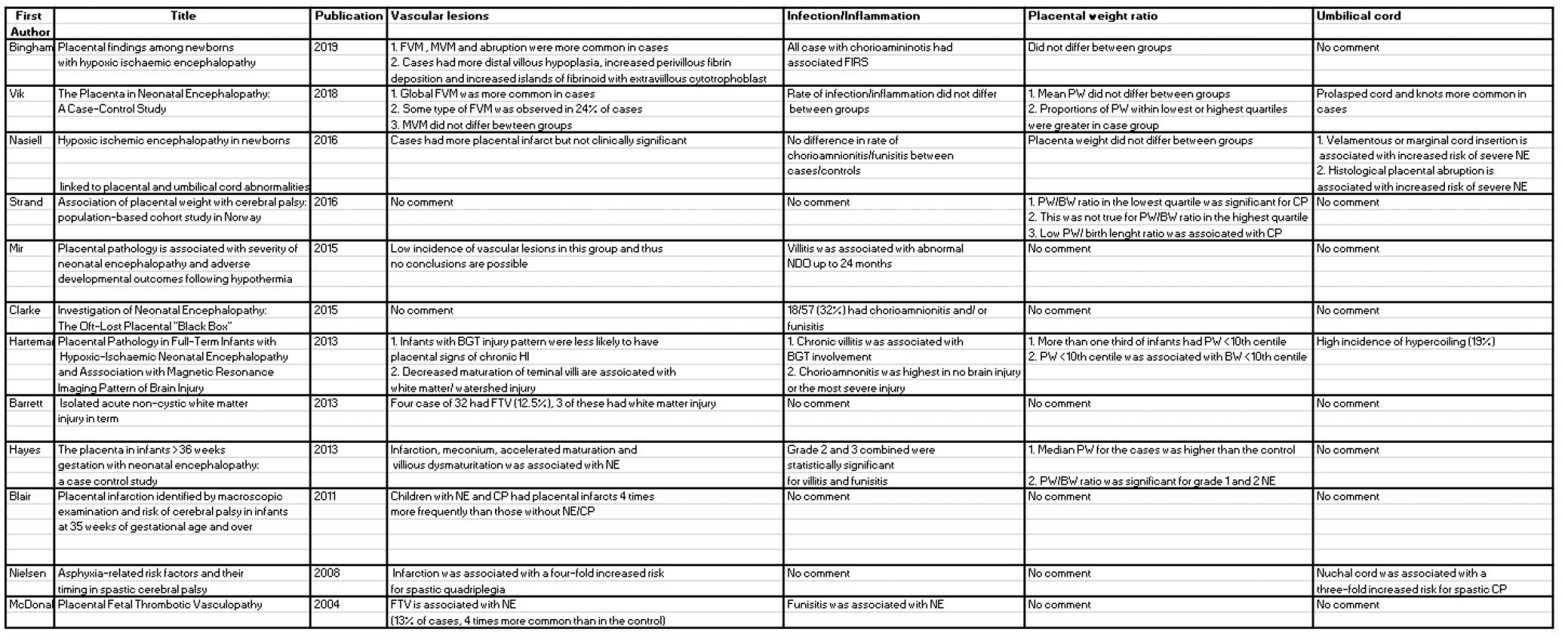
Original research articles reviewed with subheadings.

A total of 170 titles were reviewed, of which 67 abstracts were reviewed. Forty‐five publications were reviewed in full. Twenty‐seven original articles/review papers were used to compile this review article (Figures 1, 2, 3). Eighteen articles were excluded because they did not relate to the content of this review. Twelve original articles were reviewed (six case–control studies, three cohort studies, and three retrospective case reviews). Fifteen review articles were reviewed. There was heavy heterogeneity among the papers and therefore a metanalysis was not possible.

There was a high incidence of placental abnormalities associated with NE. Mir et al^9^ conducted a retrospective review of 120 babies with HIE, of whom 73 had moderate–severe encephalopathy and received therapeutic hypothermia (TH). A total of 95% of this cohort had histological placental pathology, with 65% having major pathology.^6^ Placental lesions are common; however, the odds of NE increase as the number of placental lesions increase.^15^, ^16^ Figures 1, 2, 3 outline the original and review articles used to compile this paper and include a summary of their main findings and conclusions.

It was possible to group our findings into three main themes. The main associations between NE and placental pathology in the literature relate to FVM, such as umbilical cord pathology, MVM including fetoplacental weight ratio, and inflammation.

### 3.1 Fetal vascular lesions

Fetal vascular lesions have been found to occur in 20% of infants who develop NE or who are subsequently diagnosed with cerebral palsy (CP).^8^

Redline and Ravishankar^16^ outline two distinct patterns of FVM: global partial pattern and segmental complete pattern. These two patterns can be found to coexist and the global partial pattern can progress into segmental complete pattern.

Vik et al^2^ performed a case–control study examining placental findings for babies with NE. This study found that one in five babies born with NE had evidence of FVM in the placenta. McDonald et al^1^ conducted a review of placenta and its association with NE. This study found FVM, described in the paper as “FTV,” to be a clinically significant factor in the development of NE and concluded that it was four times more common in the babies with NE than in controls.^1^

Volpe^19^ published a review article in 2019 highlighting the importance of FVM in the pathogenesis of NE. He outlined the importance of looking for early indicators of FVM (eg, specialized targeted placental magnetic resonance imaging) to enable targeted peripartum and postpartum management.

Redline and Ravishankar^16^ published a review of FVM in 2018, wherein they outline that extrinsic pressure (eg, umbilical cord entanglements and umbilical cord prolapse), intrinsic deformation of the cord (eg, hypercoiling, torsion, and true knots) and increased resistance to blood flow in the cord (eg, excessive cord length) are mechanisms that lead to obstruction and stasis within the cord. They list compounding factors and how they play a role in FVM. They also report that the risk of FVM is increased with each number of additional umbilical cord abnormalities.^16^

Nasiell et al^3^ conducted a prospective cohort study of placenta and umbilical cord insertion. This study found that placental and cord abnormalities have a “profound association” with HIE, with a particular note on umbilical cord insertion. “A velamentous or marginal cord was associated with risk of severe HIE.”

### 3.2 Maternal vascular malperfusion

Features of MVM have been found to be more prevalent in the placenta of babies born with NE as a presumed consequence of hypoxia.^8^

Placenta weight and fetoplacental ratio that are relatively high or low have been associated with placental insults and maternal hypertensive disorders. This has been shown to impact neonatal condition at birth and be associated with increased risk of CP in many studies.^2^, ^15^, ^21^, ^22^ However, Hayes et al^15^ found that high fetoplacental ratio was associated with lower incidence of grade 3 NE.

### 3.3 Inflammation

Mir et al^9^ found that chorioamnionitis (with or without an inflammatory response) and patchy/diffuse chronic villitis were independently associated with severity of encephalopathy (*P* < 0.001).^9^ They found an association between diagnosis of major placental abnormality and abnormal neurodevelopmental outcome after TH; specifically, diffuse chronic villitis was found to be an individual predictor of this finding.^9^ Many studies have found an association between chorioamnionitis and development of CP, with some studies reporting a two‐fold increased risk of CP.

### 3.4 Placental associations with CP

Blair et al^26^ conducted a population‐based case–control study investigating the relationship between CP and macroscopic placental infarction. They found that infarction alone was associated with a “tripled risk of CP” and that the mothers more often had prior miscarriages. They speculate that this could indicate an additional underlying vasculopathy which could cause harm to the “fetal cerebral vasculature or brain directly.” Studies have found that “only 13% of term babies who exhibit NE are later diagnosed with CP.”.^7^

## 4 DISCUSSION

There are multiple pathological features contributing to the pathogenesis of NE. The papers reviewed in the article delineate the main areas of importance for disease prevention. The core elements include FVM, umbilical cord insertion, MVM, placenta weight and ratios, and inflammation.

FVM is likely to be a significant factor for the 65%–75% of babies with NE who have no sentinel event. In 2018, Vik et al^2^ supported this theory. They proposed that a substantial proportion of infants with apparently acute hypoxic ischemia presumed to be peripartum in origin have a subacute or chronic abnormality of the placenta, i.e., FVM. FVM over the last few days or weeks of pregnancy causes impaired fetal blood flow and oxygenation, which can be postulated to lead to significant cerebral hypoxia‐ischemia, reduction in cerebral glycogen and energy reserves, and impairment of cerebrovascular autoregulation. This renders the fetal brain vulnerable to the normal physiological hypoxia of labor. There is currently ongoing research looking at modes of assessing FVM antenatally including through the use of ultrasound and magnetic resonance imaging to evaluate the placenta, showing promising results.^28^, ^29^ Advances in this area have the potential to allow earlier identification of the at‐risk fetus.

The most common cause of malperfusion is umbilical cord obstruction leading to stasis, ischemia, and in some cases thrombosis.^16^ Malposition of the cord leads to a vulnerability to compression and then obstruction. The impact of cord compression can be further compounded by medical issues leading to hyperviscosity. Many cases of FVM are likely to be a consequence of umbilical cord pathology or injury. Abnormal cord positioning is a significant risk factor for FVM and consequently the morbidity and mortality associated with NE. Routine identification and reporting of umbilical cord insertion could aid in the risk assessment of a fetus.^30^, ^31^ Identifying abnormal cord position would facilitate alternate pathways to delivery and help identify those who may benefit from additional screening, eg, identify medical conditions contributing to hypercoagulability.

The role of vascular lesions on the maternal side of the placenta (MVM) warrants consideration. MVM is implicated in preeclampsia, fetal death, small‐for‐gestational age (SGA) neonates and spontaneous preterm labor and premature rupture of membranes.^20^ Features include distal villous hypoplasia, increased perivillous deposition, and increased islands of fibrinoid with extravillous cytotrophoblast. It has a recurrence risk of 10%–25%. MVM has not been directly linked with NE^2^, ^9^, ^15^; however, there is evidence that SGA/intrauterine growth restriction (IUGR) neonates have impaired tolerance at labor and have a higher risk of NE relative to a well‐grown baby.^15^

MVM identified in a previous pregnancy could facilitate an augmented management plan for future pregnancies in an attempt to improve placenta function and limit the effects on fetal growth.

The relationship between placenta weight and fetoplacental ratio remains inconclusive as studies have found contrasting results. Hayes et al.^15^ proposed that placental hypertrophy in response to adversity may be protective against grade 3 NE and therefore prevent grade 3 NE. Abnormality of the fetoplacental ratio is a surrogate marker of placental dysfunction and further implicates the placenta in the NE disease process.

Inflammation of the placenta (with or without the presence of infection) has been shown to be present for many babies with NE. It is recognized that chorioamnionitis associated with infection has a role in the development of neonatal sepsis. Neonatal sepsis is not a common feature in NE and therefore the inflammation described in the placenta in cases of NE more commonly represents noninfectious inflammation in the form of chronic villitis or sterile histological chorioamnionitis.

Further evidence for the role of inflammation is the association of chronic villitis with IUGR, stillbirths, CP, neonatal seizures, and perinatal stroke in live‐born infants.^18^, ^23^ Chronic villitis, also known as villitis of unknown cause is the infiltration of the chorionic villi with maternal lymphocytes and histiocytes. This was previously thought be a result of infection with an unknown pathogen. Current evidence suggests that it represents a failure of maternal‐fetal/placental tolerance (maternal type 1 delayed‐type hypersensitivity allograft reaction).

Inflammation has gained new interest in recent years with research into the fetal inflammatory response syndrome (FIRS). FIRS is the fetal response to intra‐amniotic inflammation and manifests itself as chorionic vasculitis and funisitis (infiltration of the umbilical cord with fetal neutrophils).^24^ In the setting of infection, FIRS is thought to represent a response to exposure to infection in the previous 24–48 h. Maternal infection can lead to vertical transmission of microorganisms to the fetus. FIRS is also associated with sterile intra‐amniotic inflammation, which is likely the cause of this clinical syndrome for babies with NE. The pathogenesis for this is poorly understood.^25^ FIRS has the potential to impact the fetal/neonatal brain in several ways through its direct impact on the brain and through secondary effects on the brain from other systemic manifestations. Inflammatory cytokines are thought to cause direct damage to the developing brain through neuronal damage, impaired oligodendrocyte function, and impaired cerebral perfusion. This is further compounded by the postnatal consequences of FIRS, including cytokine cascade, coagulopathy, and systematic hypotension.^24^

The literature supports IUGR as a clinically significant risk factor for the development of NE and CP with or without prior NE.^22^, ^27^ This may represent long‐standing placental insufficiency leading to poor fetal growth in the third trimester of pregnancy. Poor fetal growth with a suboptimal environment may be the primary cause for brain injury and/or render the fetal brain vulnerable to a second insult.^17^ On the contrary, there has also been evidence to show that earlier exposure to suboptimal conditions may be neuroprotective in the event of an additional insult (negative preconditioning).^17^ Babies with IUGR/SGA are identified antenatally but perhaps screening for additional risk factors of placental dysfunction may be useful to identify the subset of growth‐restricted babies least tolerant of normal labor processes.

This paper focuses on NE; however, there is a large group of babies who may have morbidity from placenta pathology that are not evident at birth. CP is defined as a disorder of movement, posture, and motor function caused by a nonprogressive lesion or abnormality of the developing brain.^26^ CP is a well‐recognized consequence of NE and specifically NE as a consequence of hypoxia‐ischemia. Before the introduction of TH, one in three infants with moderate to severe NE were at risk for developing CP.^18^, ^23^

Only one in eight cases of CP is associated with a neonatal diagnosis of moderate to severe encephalopathy.^23^ Studies have shown that a significant number of children with CP in the absence of NE have evidence of placental pathology, specifically placental infarction.^26^ Prolonged exposure to placental malperfusion may result in chronic low‐level hypoxia causing neonatal brain injury without encephalopathy at birth.

## 5 CONCLUSIONS

This review highlights the importance of placental assessment in the investigation of NE. Placental abnormality is an important pathway priming the fetal brain for poor tolerance of labor. It is a primary or secondary cause for many cases of NE.^17^, ^18^ Identifying these factors postpartum on placental histology is helpful for counseling parents as to the likely cause. However, antenatal diagnosis may also be of benefit. Additional research in this area may facilitate both improved antenatal and postnatal care of babies with NE. Antenatal care could utilize additional screening to facilitate individualized care for at‐risk fetuses with atypical placental function or anatomy. Classifying the cause of hypoxia into acute, subacute, or chronic based on placental findings may enable stratification of postnatal therapies such as TH.

Much of the research on the placenta is based on national patient registries using macroscopic examination for analysis. This has led to deficiencies in our knowledge. Research looking at detailed placental histology could help further delineate the placenta's role in the pathogenesis of NE and thus potentially impact patient care.

The obstetric and neonatal community need to work together to use this information to improve how we identify and manage at‐risk mothers and fetuses.

## AUTHOR CONTRIBUTIONS

Aine Fox performed the literature search and wrote the main body of the article, Emma Doyle contributed to the interpretation and review of the intellectual content of the article, Michael Geary contributed to the design and editing of the article, and Breda Hayes contributed to the concept, design, and editing of the article.

## CONFLICT OF INTEREST

The authors have no conflicts of interest.

## Data Availability

Data sharing is not applicable to this article as no new data were created or analyzed in this study.
